# Effects of asymmetrical postural demands on sternocleidomastoid reflex in the startReact paradigm

**DOI:** 10.3389/fnhum.2025.1592691

**Published:** 2025-09-23

**Authors:** Ermyntrude N. A. Adjei, Kelsey Wright, Julius P. A. Dewald, Jun Yao

**Affiliations:** ^1^Department of Biomedical Engineering, Northwestern University, Evanston, IL, United States; ^2^Department of Physical Therapy and Human Movement Sciences, Northwestern University, Chicago, IL, United States; ^3^Northwestern University Interdepartmental Neuroscience, Evanston, IL, United States; ^4^Department of Physical Medicine and Rehabilitation, Northwestern University, Chicago, IL, United States

**Keywords:** sternocleidomastoid, startReact, postural demands, reticulospinal tract, anticipatory postural adjustments, shoulder abduction, hand

## Abstract

**Introduction:**

startReact, the rapid release of a planned movement following a startling acoustic stimulus (usually >100 dB), is widely used to assess reticulospinal tract (RST) involvement in motor control. The sternocleidomastoid (SCM) reflex within 120 ms often identifies true startle responses, i.e., responses facilitated by RSTs in the absence of cortical control. However, as the SCM is a postural muscle, its reflexive activation may be influenced by inhibitory anticipatory postural adjustments (APAs), particularly during tasks with greater head/neck postural demands.

**Methods:**

We compared SCM activation during unilateral shoulder abduction (SABD) versus hand opening (OPEN) tasks. Due to the increased asymmetrical head/neck postural demands in SABD, we hypothesized an APA-induced delay in SCM activation during SABD compared to the OPEN task, with a contralateral bias due to contralateral cortical circuits triggering APAs.

**Results:**

Our results revealed significantly longer SCM latency—exceeding the 120 ms cutoff—during SABD relative to OPEN. This suggested that APAs during postural tasks, resulting from unilateral SABD, altered the expression of the startReact response. To confirm this finding, we implemented an innovative, data-driven method to determine the appropriate SCM cutoff based on the physiological difference between startle-induced SCM reflexes and task-induced SCM activation. Using this method, we observed reduced contralateral SCM reflexive activation compared to ipsilateral, during SABD but not in OPEN.

**Discussion:**

This provides evidence for the first time that SCM reflexive activation in startReact is posture-dependent. Our novel classification method offers a robust framework for identifying true startle responses across different tasks, offering broader applicability for studies investigating RST involvement in motor control.

## Introduction

In humans, upper extremity (UE) movements involve a task-dependent interplay between corticospinal and reticulospinal tracts (CSTs and RSTs) (Baker, 2011; Welniarz et al., 2017). Despite insights from non-human primate studies (Lawrence and Kuypers, 1968), the roles of these pathways in human UE motor control remain unclear. A prominent non-invasive method to investigate the role of RSTs is the startReact paradigm, which uses a startling acoustic stimulus (SAS, > 100 dB) to trigger the rapid release of a pre-planned movement. The SAS can trigger either a “false startle” (SR−) or a “true startle” (SR+) response. The SR− response is a fast voluntary reaction influenced by cortical processing and facilitated via transcortical pathways (Maslovat et al., 2015). In contrast, during an SR+ response, SAS activation of the cochlear nucleus excites the reticular formation (RF), the origin of the RSTs, bypassing cortical processing and leading to a rapid, involuntary release of the planned movement via the RSTs (Yeomans and Frankland, 1995; Tapia et al., 2022; Lingenhöhl and Friauf, 1994). Such RF excitation also induces sternocleidomastoid (SCM) reflexive activation typically within ∼120 ms of SAS (Brown et al., 1991b), which is widely used to distinguish SR+ from SR− responses (Carlsen et al., 2007; Honeycutt et al., 2013). However, as the SCM is a postural neck muscle, its use as an SR+ indicator may be compromised in tasks with increased postural demands, hindering investigations of RST use in such tasks.

Sternocleidomastoid receives bilateral corticobulbar inputs that contribute to head and neck posture through anticipatory postural adjustments (APAs) preceding movement onset, among other postural adjustments (Bhardwaj and Yadala, 2023; Takakusaki et al., 2017). For example, SCM amplitude is significantly reduced during known or self-induced perturbations compared to unexpected ones (Blouin et al., 2003; Kuramochi et al., 2004; Bartsch-Jiménez and Valero-Cuevas, 2025), reflecting the effect inhibitory APAs have on SCM activation. This APA-induced SCM suppression also manifests as delayed responses, as prior knowledge of an impending postural perturbation delays peak head acceleration (Kumar et al., 2000). Due to these challenges, previous startReact studies have primarily focused on tasks with minimal APA demands, such as distal joint movements (Honeycutt et al., 2013; Carlsen et al., 2009) or bilateral SABD (Maslovat et al., 2023). As a result, tasks like unilateral shoulder abduction (SABD), which especially rely on RSTs (Davidson and Buford, 2006) and exhibit strong APA-induced SCM modulation (Falla et al., 2004) remain underexplored.

Our primary objective was to investigate the effect of such proximal postural tasks on the SCM reflexive activation in startReact paradigms. We compared SCM activation in startReact between a right-arm shoulder abduction (SABD) task and a right-hand opening (OPEN) task in right-hand-dominant participants. The OPEN task served as a comparison due to its minimal postural demands while still eliciting a true startle response (Honeycutt et al., 2015). We hypothesized that the increased asymmetrical neck postural demands associated with the unilateral SABD task (Falla et al., 2004), would necessitate APA-induced SCM suppression, leading to delayed SAS-induced SCM activation, beyond the 120 ms cutoff, compared to the OPEN task. This delay could increase false negatives when using the 120 ms cutoff to distinguish between SR+ and SR− responses.

To address the limitations of the 120 ms cutoff, we employed a data-driven approach based on the physiological difference between true startle-induced SCM reflexes and task-induced false startle SCM activation. This approach allowed us to show that any observed task differences in SCM reflexive activation were due to the APA inhibitory effect. Studies by Caronni and Cavallari (2009a,b) have demonstrated an inhibition of motor-evoked potentials from the contralateral hemisphere within the APA window prior to movement onset. This result was without corresponding changes in the spinal reflexes of postural muscles, suggesting that contralateral cortical, not spinal, circuits drive inhibitory APAs. Additionally, transcranial stimulations have shown that cortical projections from the contralateral hemisphere to the SCM are stronger and faster than those from the ipsilateral hemisphere (Gandevia and Applegate, 1988; Thompson et al., 1997). We thus hypothesized that an APA inhibitory effect on SCM reflexive activation, would manifest as a laterality difference with more pronounced suppression in the right SCM (the muscle contralateral to the active motor cortex) than left SCM. This would be evident as a reduced amplitude and a longer delay in the right SCM during the SR+ condition in the SABD task. Such laterality difference is not expected during the OPEN task.

Unlike previous studies that focused on postural tasks with minimal head/neck perturbation or lower-limb tasks (MacKinnon et al., 2007; Brown et al., 1991a; Ravichandran et al., 2013; Heckman and Perreault, 2019), we directly impacted head/neck postural demands and observed their influence on SCM activation in startReact. Given that startReact is the most widely used non-invasive tool to assess RST’s role in motor control, our findings provide novel evidence of the effect of increased asymmetrical postural demands on startReact’s true startle response indicator, the SCM. For the first time, we show that SCM activation in the startReact paradigm is not solely driven by startle-triggered reflexes but is influenced by head/neck postural demands. These results call for a refinement of the startReact methodology used in advancing our understanding of RST involvement in motor control.

## Materials and methods

### Participants

Twenty-nine adults [eight males, aged mean (SD): 31.8 (12.7) years], who were right-hand dominant as determined by the Edinburgh Handedness Inventory (> 61%), participated in the study. None of the participants had any history of neurological or musculoskeletal disease or exhibited hearing loss (hearing threshold less than 30 dB). The pure-tone audiometry, a standardized clinical hearing test, was used to assess each participant’s hearing threshold levels. This test evaluates the softest sound a person can detect at specific frequencies, typically between 250 and 8,000 Hz (Musiek et al., 2017). We focused at 500, 1,000, and 2,000 Hz for both ears. While all participants met the criteria for all three frequencies, our statistical analysis was focused on 2,000 Hz, as the lowest sound intensity during the experiment occurred at this frequency. A paired *t*-test revealed that there was no significant difference in the hearing threshold between the left and right ears across participants at 2,000 Hz [t(27) = 0.75; *p* = 0.46]; mean difference (95% confidence interval) = 0.7 (−1.2, 2.7) dB, Left ear mean (SD): = 7.1 (8.3) dB; Right ear mean (SD): = 7.9 (7.1) dB. See Table 1 for the demographic information for each participants.

**TABLE 1:** Participant parameters, showing the age, sex, hearing threshold, and the handedness test.

Participant	Age	Sex	Hearing threshold (dB)		Handedness (%)
			Right ear	Left ear	
1	48	F	10	10	100.0
2	24	F	5	0	62.5
3	24	M	25	20	100.0
4	22	F	10	10	75.0
5	40	F	10	10	87.5
6	25	F	15	15	87.5
7*	30	F	< 30	< 30	100.0
8	54	F	5	5	100.0
9	23	F	0	5	100.0
10	25	F	5	0	100.0
11	50	F	15	15	100.0
12	26	F	5	5	100.0
13	23	M	0	0	100.0
14	64	F	25	25	100.0
15	19	F	10	10	100.0
16	20	F	5	5	87.5
17	35	M	5	5	100.0
18	28	M	5	0	100.0
19	32	M	0	0	87.5
20	29	M	10	10	100.0
21	18	F	10	10	100.0
22	30	F	10	5	100.0
23	29	F	5	−5	100.0
24	64	F	20	30	100.0
25	31	M	5	5	100.0
26	22	F	0	0	100.0
27	25	F	−5	10	100.0
28	41	F	5	0	100.0
29	22	M	5	−5	87.5

The hearing threshold represents the lowest air conduction sound the participant could detect at 2,000 Hz frequency. The handedness represents the results from the Edinburgh Handedness Inventory Short Form.

*Participant seven was marked as eligible; however, without specific hearing threshold documented.

### Ethical approval

This study was approved by the Northwestern institutional review board (STU00212195) and all participants provided their written informed consent that conformed to the standards set by the latest revision of the 1964 Helsinki Declaration and its later amendments, except for registration in a database.

### Experimental design

**FIGURE 1:**
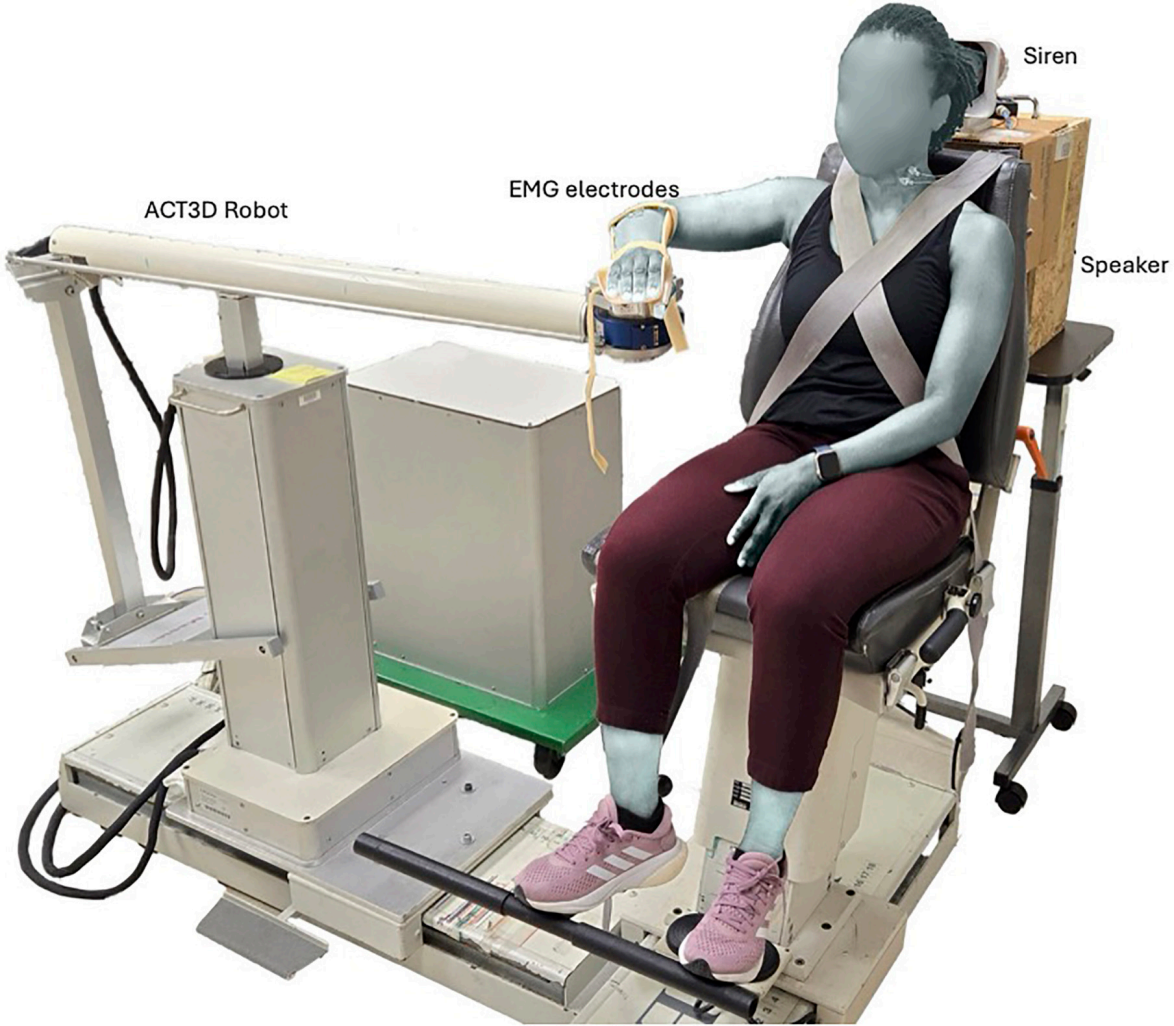
Experimental set-up illustrating baseline arm positioning on the ACT3D robot, as well as the siren and speaker used to generate the STARTLE and CONTROL cues, respectively.

Each participant was seated in a height-adjustable Biodex chair (Biodex Medical Systems, Shirley, NY), positioned approximately 2 m away from a 40 inch LCD computer monitor. They sat without a headrest, and with their trunk and shoulder securely strapped in place to prevent trunk postural adjustments and motion during experiments (Figure 1). Their right hand and forearm were placed in an orthosis attached to the end effector of an Arm Coordination Training 3-D admittance- controlled robot (ACT-3D - HapticMASTER™, Motekforce Link, DIH Medical group, Amsterdam, The Netherlands). The ACT-3D robot was instrumented with a six-degree-of-freedom load cell, capable of generating and recording perpendicular forces to the horizontal plane and allows for 3D arm movements.

Prior to the experiment, the maximum voluntary force of the right arm in the direction of SABD was recorded under configuration of 90-degree shoulder abduction, 40-degree shoulder flexion, and 90-degree elbow flexion.

Participants were required to perform two motor tasks — SABD and OPEN, using their right arm. At the start of each trial, each participant’s arm rested on a virtual table with the arm in the home position. This position corresponded to a configuration of 85-degree shoulder abduction, 40-degree shoulder flexion, and 90-degree elbow flexion (Figure 1). Since the arm was fully supported by the ACT3D robot, which generated a virtual haptic and frictionless table, we referred to this horizontal plane as the virtual table. For the SABD task, participants were instructed to abduct their right arm above the virtual table while exerting a force equivalent to 53% of their maximum SABD strength. While a displacement range of 5–10 degrees was suggested, the primary requirement was that participants lifted off the table. This lift-off was visually monitored during data collection to ensure consistent performance across trials. The 53% load was selected because it was the heaviest resistance used in a related study, where shoulder abduction load was increased from 25% to 53% of their maximum in 7% increments (Wright et al., 2024). In Wright’s study, the 53% load was chosen as the maximum load to avoid fatigue, as the task required 28–30 repetitions per load. In this present study, we adopted this maximum load to maximize APAs necessary for investigating the effect of increased head/neck postural demands on SCM activation. This choice of SABD with 53% load not only ensured increased head/neck postural demand, but also targeted a movement primarily facilitated by the RSTs (Sukal et al., 2007; Davidson and Buford, 2006). For the OPEN task, participants were instructed to perform a maximal right hand opening with their right arm relaxed on the virtual table.

Each participant was trained to self-initiate the task after a fixed preparation phase of either 5 or 6 seconds, following an 80 dB warning cue (5–8 training trials). This fixed interval was chosen to ensure maximal motor preparation, as the present study was part of a larger investigation into cortical and brainstem contributions during motor preparation for voluntary movements. Training continued until they could consistently self-initiate the required task within ± 0.5 s of the target timing for three consecutive trials, without relying on counting. Participants received similar training for each task.

All participants were also instructed that if they heard a second cue during the preparation phase, they should perform the required task as quickly as possible. This second cue was either a non-startling sound (CONTROL cue – 80 dB, 2 kHz, 40 ms duration, via a speaker) or a STARTLE sound [120 dB, frequency range (0.4–4 kHz), 100 ms duration, via a siren (model M85PDS)]. A broader frequency range of the startling sound was chosen because such stimuli are more likely to elicit SR+ responses than a fixed tone (Carlsen, 2015). The sound intensity was confirmed using a sound meter (BAFX3370, Digital Sound Level Meter) placed at the participant’s ear.

During the experiment, each task was completed in a block consisting of 28–33 trials. The first three trials were always without a second cue. These self-initiating trials were used to confirm that participants performed the tasks as required. In 70%–80% of the rest of trials in a block, a second cue was randomly delivered between 3.5 and 0.5 s before the required preparation time (CONTROL cue – 14 trials per task, STARTLE cue, 6–8 trials per task. This range was to ensure that the SAS was delivered during a high state of preparation while preventing temporal predictability (MacKinnon et al., 2013). Importantly, this timing window did not affect SCM latency or reaction time. For the rest 20%–30% of trials in a block, self-initiating trials were interspersed in a pseudorandomized order, ensuring that no two consecutive trials were designated as STARTLE trials. These self-initiating trials, together with the variable fore-period between warning and second cues, ensured that participants were actively planning to execute the task without anticipation and prevented false starts (Carlsen et al., 2004, 2007, 2009). Fifteen of the 23 participants began with the OPEN task, as this task served as an exclusion criterion to ensure participants could exhibit a SR+ response under typical startReact paradigms (Honeycutt et al., 2015).

We recorded electromyographic (EMG) data from the intermediate deltoids (IDL), extensor digitorum communis (EDC), and the bilateral sternocleidomastoids (SCM). For each targeted muscle, two flat active monopolar Biosemi electrodes (Active II; Biosemi, Inc., Amsterdam, The Netherlands) were placed on the muscle belly, along the direction of muscle fibers. All the monopolar electrodes shared a common reference electrode, positioned over the mastoids. In addition, we recorded the maximum voluntary muscle activation (MVC) for the IDL and EDC at the home position under isometric conditions for normalization purposes.

### Data analysis

We first calculated the differential bipolar EMG by calculating the difference in electrical potential between each pair of monopolar electrodes from the same muscle. The resulting bipolar EMG signals were then filtered using a 4th order high-pass Butterworth filter with zero phase shift and a cut-off frequency at 20 Hz. The EMG onset was automatically identified as the first point where the filtered EMG signal exceeded three times the standard deviation from baseline, which was determined from a 100 ms window preceding the second cue. This was then visually inspected and manually corrected as required.

We measured the SCM latency as the time interval between the onset of the STARTLE cue and the earlier onset of the two SCMs. This was restricted to the STARTLE cue as SCM activation was nearly absent in the CONTROL condition during the OPEN task. We also calculated the reaction time (RT) as the time interval between the onset of the second cue and the onset of the primary muscle driving each task. Specifically, the EDC and IDL were used as primary muscles for the OPEN and SABD tasks, respectively. Trials with RT less than 40 ms or greater than 500 ms were regarded as outliers and excluded from further analysis. These accounted for less than 2% of all trials.

Furthermore, we employed a z-score normalization technique to normalize the amplitudes of both of the SCM muscles using the equation 1 below.


(1) $$\begin{array}{l} Amplitude_{Z-NORM}=\frac{\left(relativ\;eactivation-mean\right)}{standard\;deviation}\\ where,\;relative\;activation\;Amplitude-Baseline\;activity \end{array}$$

The mean and standard deviation were computed across the two SCMs, ensuring consistent values for both the right and left SCMs. Baseline activity and the amplitude were calculated as the root-mean-squared value (RMS), within 100ms before and after the onset of SCM activation, respectively.

### Categorizing SR+ vs. SR− responses

Instead of using the conventional arbitrary cut-off at 120 ms, to distinguish between true (SR+) and false (SR−) startle response trials, we employed a data-driven approach based on two key criteria: (1) the relationship between RT and the SCM latency reflecting the physiological distinction between true startle-induced SCM reflexes and task-induced SCM activation, and (2) SCM amplitude in the STARTLE trials. The startReact circuitry posits that startle-induced activation of the reticular formation (RF) could simultaneously trigger the activation of the accessory nucleus, due to the excitation of reticular neurons in close proximity (Davidson and Buford, 2006; Brown et al., 1991b). Consequently, during a SR+, where there is minimal input from the cortex (Yeomans and Frankland, 1995; Tapia et al., 2022), the parallel engagement of the RSTs and the accessory nerve (Maslovat et al., 2012) is expected to result in minimal or no correlation between RT and SCM latency. Conversely, in an SR− response with greater cortical input, the task- induced SCM activation is expected to align closely with the onset of the primary muscle activation, resulting in a linear relationship between RT and SCM latency. We modelled the relationship between RT and SCM latency by fitting the data to a sigmoid curve using the “nlraa” package in R (equation 2).


(2) $$f\,\left(x\right)=a+\frac{b}{1+e^{-c\,\left(x-d\right)}}$$

where, *x* is the SCM latency, *a* and *b* represents minimum and maximum RT, respectively and *c* determines the initial slope of the curve (starting at 0.1) and *d* is the median SCM latency. The data used consisted of all participants who exhibited measurable SCM activation in response to the STARTLE cue. This was done to ensure the sigmoid fit captured the full range of responses across participants, thereby enhancing the generalizability and robustness of the derived threshold across both tasks.

The estimated zero slope of the sigmoid represented the RST-mediated SR+ responses, while the linear portion described the cortically-influenced SR− responses. We estimated the inflection point, marking the transition between the SR+ to SR− responses as the cutoff for SR+ responses.

To quantify uncertainty in our estimates, we employed bootstrapping by resampling the data 1,000 times with replacement to generate multiple bootstrap samples. For each sample, we refitted the sigmoid model and recalculated its parameters. This yielded a distribution of inflection points, from which we derived 95% confidence intervals, providing robust estimates of the uncertainty around the cutoff.

Given that SCM activation was also observed during CONTROL (non-startle) trials, particularly in the SABD tasks, a secondary criterion for SR+ classification was introduced. A trial was considered SR+ if the amplitude of either SCM exceeded the upper confidence interval limit observed in CONTROL trials. Trials that failed to meet any one of these two criteria were categorized as SR− trials. To enable within-subject comparison in both SABD and OPEN tasks, only participants who had at least one SR+ trial in each task were included in the analysis to test the SCM laterality hypothesis. Applying this criterion reduced the dataset to the first 23 participants listed in Table 1. Of these 23, only three participants had a single SR+ trial across both tasks, indicating that low trial counts represented a minimal proportion of the data.

### Statistical analysis

We first tested the effect of Task on earlier-activated SCM latency, using a linear mixed effect (LME) model with Task (OPEN and SABD) as the fixed factor and the latency of the earlier-activated SCM as the outcome measure. Furthermore, to test that SABD would show a significantly higher probability of SCM activation occurring beyond the 120 ms cutoff compared to OPEN, we used a generalized linear mixed-effects model (GLMM) with a binomial distribution. The model included Task as a fixed effect, with the binary outcome of exceeding the cutoff (1 if SCM latency > 120 ms, 0 otherwise) as the outcome measure. The log of the total number of trials for each participant-task combination was incorporated as an offset to account for varying trial numbers, since in some STARTLE trials, no SCM activation was observed.

Using our novel data-driven criteria for identifying SR+ responses, we evaluated whether both OPEN and SABD tasks remained susceptible to startReact. This analysis aimed to assess whether our new criteria reliably identified SR+ responses in a manner consistent with previous findings (e.g., Honeycutt et al., 2015). Additionally, we examined whether the SABD task exhibited greater susceptibility to startReact, given its strong facilitation via the RSTs (Davidson and Buford, 2006). We therefore used an LME model to test whether there was a significant difference in RT between SR+ and SR− conditions within each task and whether this condition difference was larger in the SABD task compared to the OPEN task. Our model included both Condition (SR− and SR+) and Task (OPEN and SABD) as fixed factors with the outcome variable as primary muscle RT. CONTROL trials were not included in the analyses as the critical comparison was between SR− and SR+ trials (Maslovat et al., 2023). To ensure enough variance for accurate statistical comparison, only participants who had at least one trial in both tasks and both conditions were included in this analysis.

We also assessed whether the probability of eliciting a SR+ response was significantly different between SABD and OPEN tasks. This analysis tested whether the presence of an SR+ response was expressed differently depending on the task. To do this, we first applied an arcsine square root transformation to the binomial probability data, converting it into an approximately normal distribution. We then conducted a Welch t-test to account for the unequal variances.

We then employed two separate LME models to evaluate the effect of increased APA demands on the SCM laterality in the SR+ condition: one model with the outcome measure as Latency and the other as Amplitude_*Z–NORM*_. For both models, we used Task (OPEN and SABD), Side (SCM_*R*_ and SCM_*L*_), and their interaction as fixed factors.

In all models, we incorporated random intercepts for participants to account for individual variations. Additionally, a random intercept for the order in which tasks were performed, to control for potential confounds due to task sequencing as OPEN was the first task for most participants. Model comparisons showed that including task order significantly improved model fit (AIC (with order as random intercept vs. without): 3468.9 vs. 3487.9; BIC: 3487.8 vs. 3503.0; log-likelihood: –1729.5 vs. −1,739.9; χ^2^(1) = 20.97, *p* = 4.68 × 10^6^), supporting its inclusion in the final model.

In all statistical analyses, the significant level was set to *p* < 0.05. Results with p values between 0.05 and 0.1 were reported as close to significant or trends. Data were inspected for approximate normal distribution of residuals. All statistical analyses were conducted using R Programming software with LME and GLMM with binomial distribution performed using “lme4” and “glmmTMB” packages, respectively. Post hoc or contrast analyses were performed using the “emmeans” package in R and Tukey’s correction for multiple pairwise comparisons. To test for SCM laterality differences, we focused exclusively on the contrast analyses comparing the right and left SCM muscles within each task.

## Results

### SCM latency is delayed in SABD task compared to OPEN task

**FIGURE 2:**
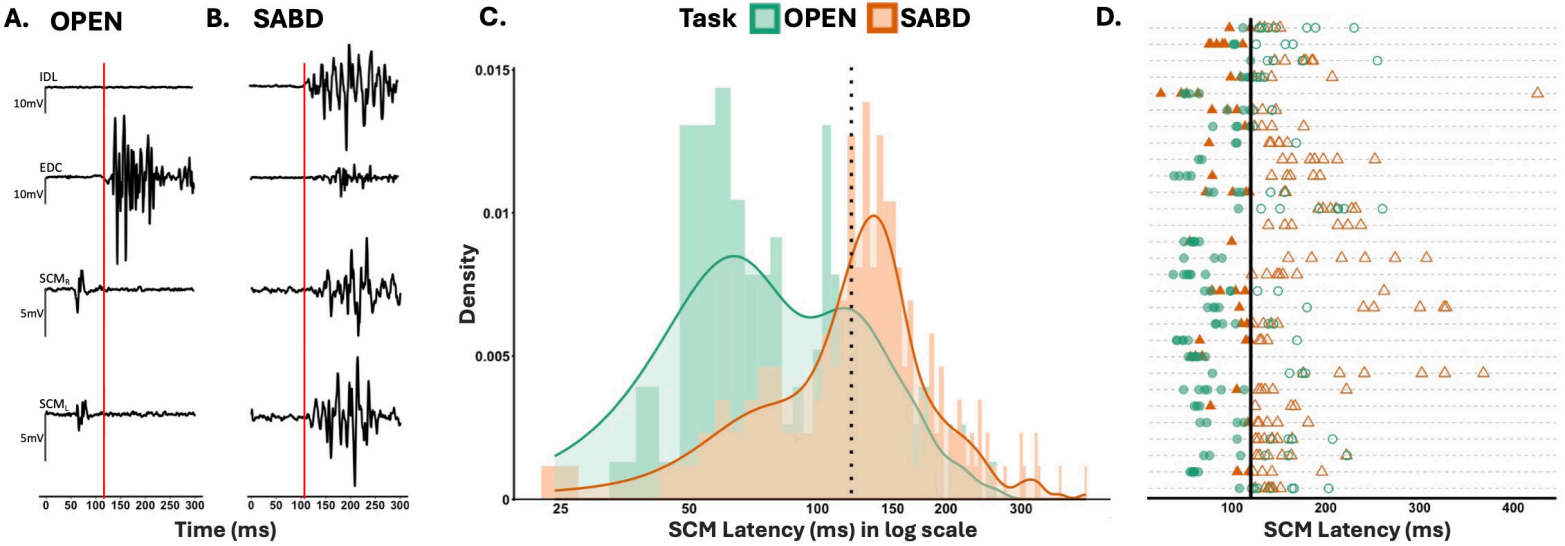
**(A,B)** Depict sample electromyographic (EMG) data of a startle response trial from hand opening (OPEN), and shoulder abduction (SABD) tasks, respectively. STARTLE cue occurred at zero. Red vertical line indicates the onset of extensor digitorum communis (EDC) and intermediate deltoids (IDL) activation for OPEN and SABD, respectively. **(C)** Illustrates the group results as a histogram of the earlier sternocleidomastoids (SCM) latency for all STARTLE trials across all 29 participants illustrated on a logarithmic scale. Dotted vertical line represents the conventional 120 ms SCM latency cutoff. Bin size = 5. **(D)** depicts each participant’s SCM Latency for all STARTLE trials. Each line represents participant data. Filled points (SCM latency < 120), unfilled points (SCM latency > 120). OPEN (green circles), SABD (orange triangles). Solid vertical line represents the conventional 120 ms cutoff.

Figures 2A, B illustrate sample EMG data of a STARTLE trial for unilateral OPEN and SABD, respectively from a representative participant. These reveal a delay in SCM activation during the SABD task, compared to that during the OPEN task. Group results demonstrated that the SCM latency delay in the SABD task compared to the OPEN task was consistent across all 29 participants: F(1,154) = 12.5; *p* < 0.001 (Figure 2C). Furthermore, results from the binomial GLMM revealed a significantly greater probability of SCM latency exceeding the conventional 120 ms cutoff in the SABD task compared to the OPEN task (Figure 2D): Estimated coefficient (β) = 1.4, SE = 0.4, z statistic = 3.1; *p* = 0.002. The odds of exceeding the 120 ms cutoff were 4.2 times higher in the SABD task than in the OPEN task [95% CI: (1.71, 10.1)].

### Determination of the SR+ response

**FIGURE 3:**
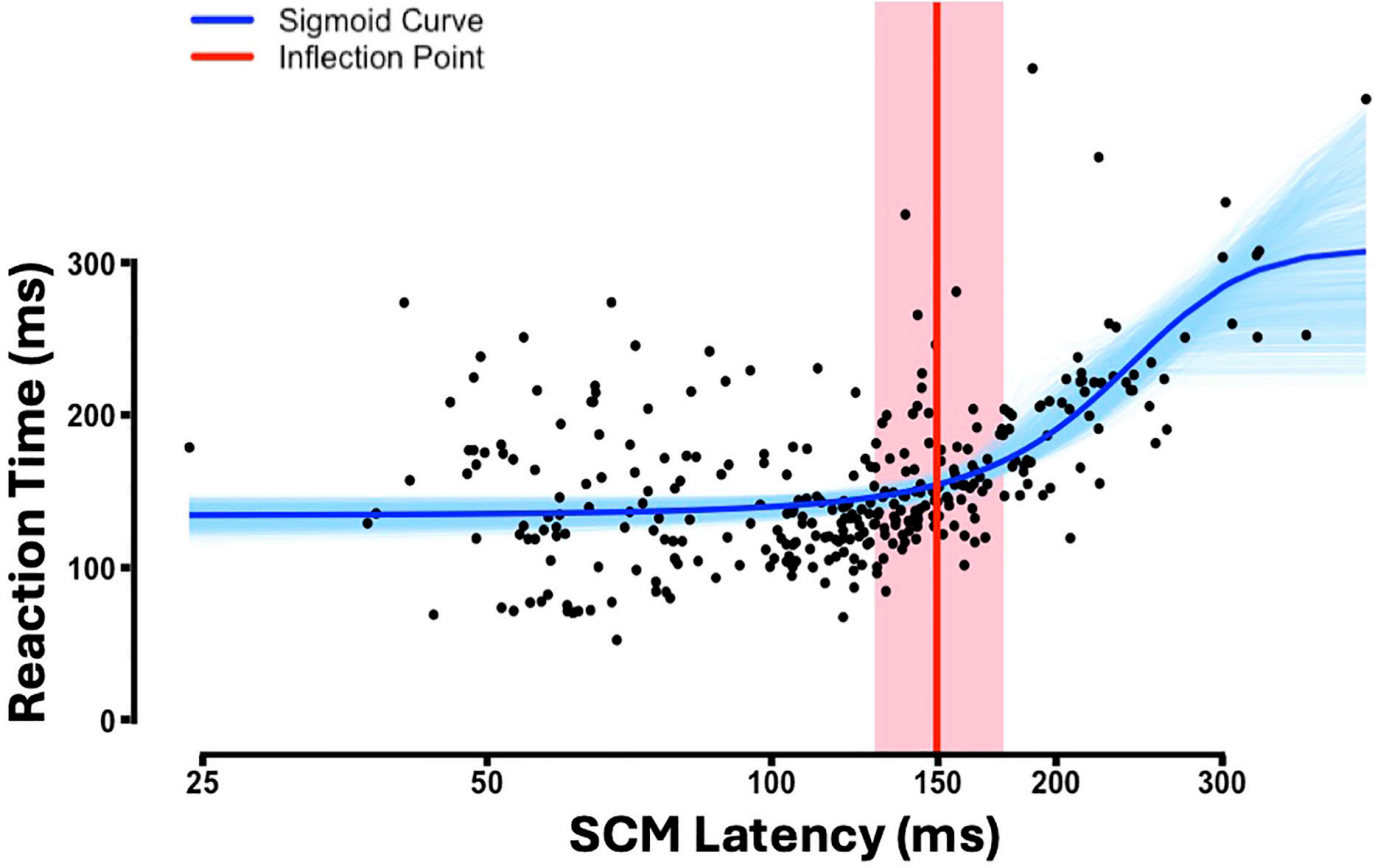
The reaction time (RT) against sternocleidomastoid (SCM) latency for all STARTLE trials (*n* = 29). Please note: the x-axis is illustrated on a logarithmic scale. Fitted sigmoid curve (blue), bootstrapped sigmoid curves across 964 iterations (light blue), estimated inflection point (red), 95% bootstrapped confidence interval for the inflection point (shaded red region).

Using the conventional cutoff would go contrary to existing literature that show that RSTs facilitate shoulder muscles relatively more than distal extensors (Davidson and Buford, 2006). Therefore, to determine a more appropriate SCM cut-off that reduces the number of false negatives, we employed a data-driven approach by assessing the relationship between reaction time (RT) and SCM latency (see section “Materials and methods”). Figure 3 illustrates the relationship between RT and SCM onset latency, fitted to the sigmoid curve. Of the 1,000 bootstrap samples, 36 (3.6%) did not converge and were excluded from the confidence interval estimation. The inflection point, representing the transition from zero to positive slope, was estimated at 149.6 ms (approximately 150 ms) with a 95% bootstrapped confidence interval of (128.7, 176.0) ms. While this range reflects some variability, the average of the bootstrapped inflection point estimates (152.9 ms) closely aligns with the inflection point estimated from the full dataset. Given this close agreement, we used the full-sample inflection point as the classification threshold. As a result, a criterion of SCM latency ≤ 150 ms, was established for categorizing trials as SR+ trials. Additionally, incorporating the SCM amplitude criterion, six participants—specifically subjects 24–29 in Table 1—were excluded from further analysis due to the absence of an SR+ response in at least one of the tasks. Using this new criterion, 3.5% of CONTROL trials during the SABD task had a SCM latency of less than 150 ms.

### SABD task is more susceptible to startReact than OPEN task: evidence from reaction time results

**FIGURE 4:**
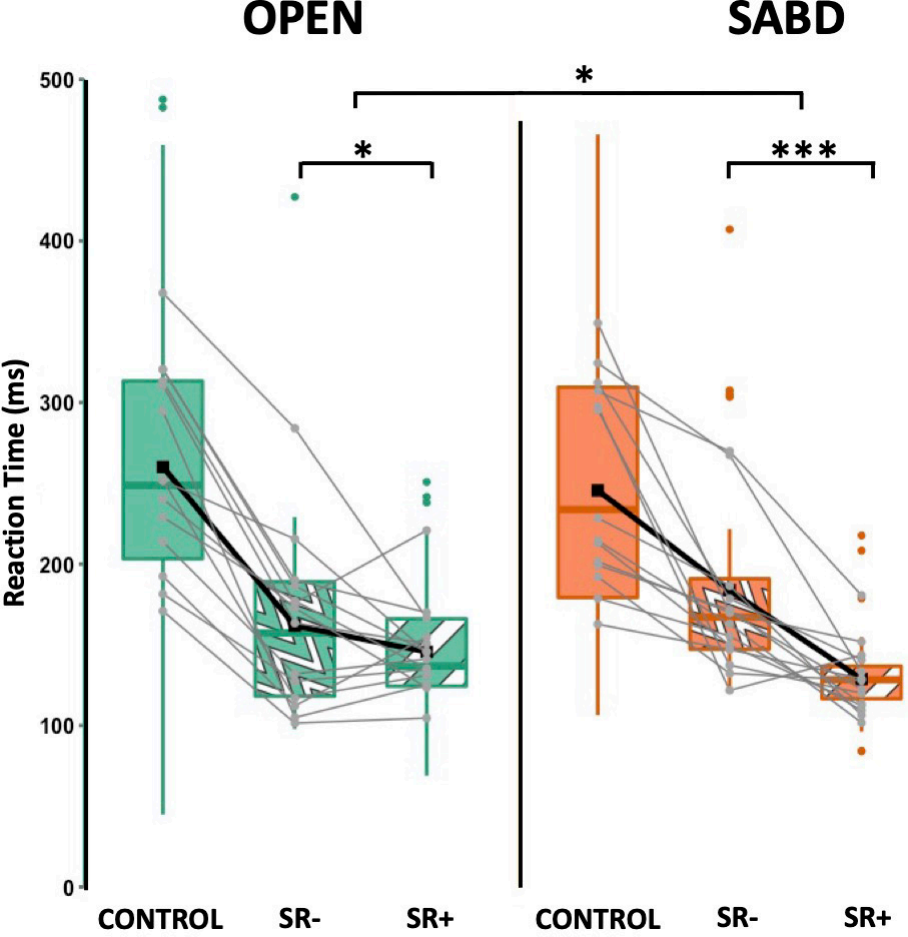
The reaction time (RT) for hand opening (OPEN) (green) and shoulder abduction (SABD) (orange) tasks under CONTROL (solid), SR– (wave patten) and SR+ (stripe pattern). Top, middle, and bottom lines of the box plot indicate the 75th percentile (top quartile), 50th percentile (median), and 25th percentile (bottom quartile), respectively. Black connecting squares in box represent the arithmetic mean. Round gray connecting dots represent the mean of each participant while the colored dots are outliers. *n* = 14. *p < 0.05, ***p < 0.001.

Using our established SR+ criteria, Figure 4 depicts the primary muscle reaction time (RT) under the CONTROL, SR− and SR+ conditions for OPEN (green) and SABD (orange) tasks. While there was no significant main effect of Task, [F(1,162) = 0.2; *p* = 0.67], there was a significant main effect of Condition [F(1,167) = 30.7; *p* < 0.001]. Additionally, we observed a significant interaction effect [F(1,165) = 6.0; *p* = 0.02]. *Post hoc* contrast analyses revealed that both tasks were susceptible to startReact, with RT in the SR+ condition being significantly shorter than in the SR− condition for both tasks. However, the condition difference was significantly larger in the SABD task compared to the OPEN task (Figure 4). The contrast analyses from the LME model are listed in the top part of Table 2. Note: Similar to previous startReact studies (Maslovat et al., 2023), CONTROL trials were not included in all statistical analyses as the critical comparison was between SR- and SR+ trials.

**TABLE 2:** Estimates from linear mixed effect contrast analyses for primary muscle reaction time (RT) (top), sternocleidomastoids (SCM) latency (middle) and amplitude_Z–NORM_ (bottom).

Primary muscle RT (ms)					
Task	SR−		SR+		*P*-value
	Mean	95% CI	Mean	95% CI	
OPEN	163.0	136.0–190.0	144.0	118.0–171.0	0.02*
SABD	181.0	156.0–205.0	132.0	108.0–156.0	< 0.001***
**SCM latency (ms)**					
**Task**	**SCM_R_ (contralateral)**		**SCM_L_ (ipsilateral)**		***P*-value**
	**Mean**	**95% CI**	**Mean**	**95% CI**	
OPEN	105.0	86.1–124.0	107.0	88.7–126.0	0.46
SABD	127.0	109.1–145.0	121.0	102.7–139.0	**0.09**
*P*-value	< 0.001***		0.02*		
**Amplitude_Z–NORM_**
OPEN	−0.42	−0.74 to −0.90	−0.44	−0.78 to −0.11	0.84
SABD	0.06	−0.22 to 0.35	0.63	0.35–0.92	< 0.001***

*P* < 0.1 (close to significant),

*p < 0.05 and ***p < 0.001. Bold values represent *p*-value < 0.1.

### Probability of SR+ responses

*T*-test results showed that there was no significant difference in the probability of eliciting a SR+ response between the SABD and the OPEN tasks: t(22) = −1.3; *p* = 0.21; mean difference (95% confidence interval) = −0.09 (−0.25 0.06). Probability mean (SD) for the two tasks were: SABD = 0.69 (0.28), OPEN = 0.75 (0.23).

### Effect of increased APA demands on the laterality of SCM reflexive activation

**FIGURE 5:**
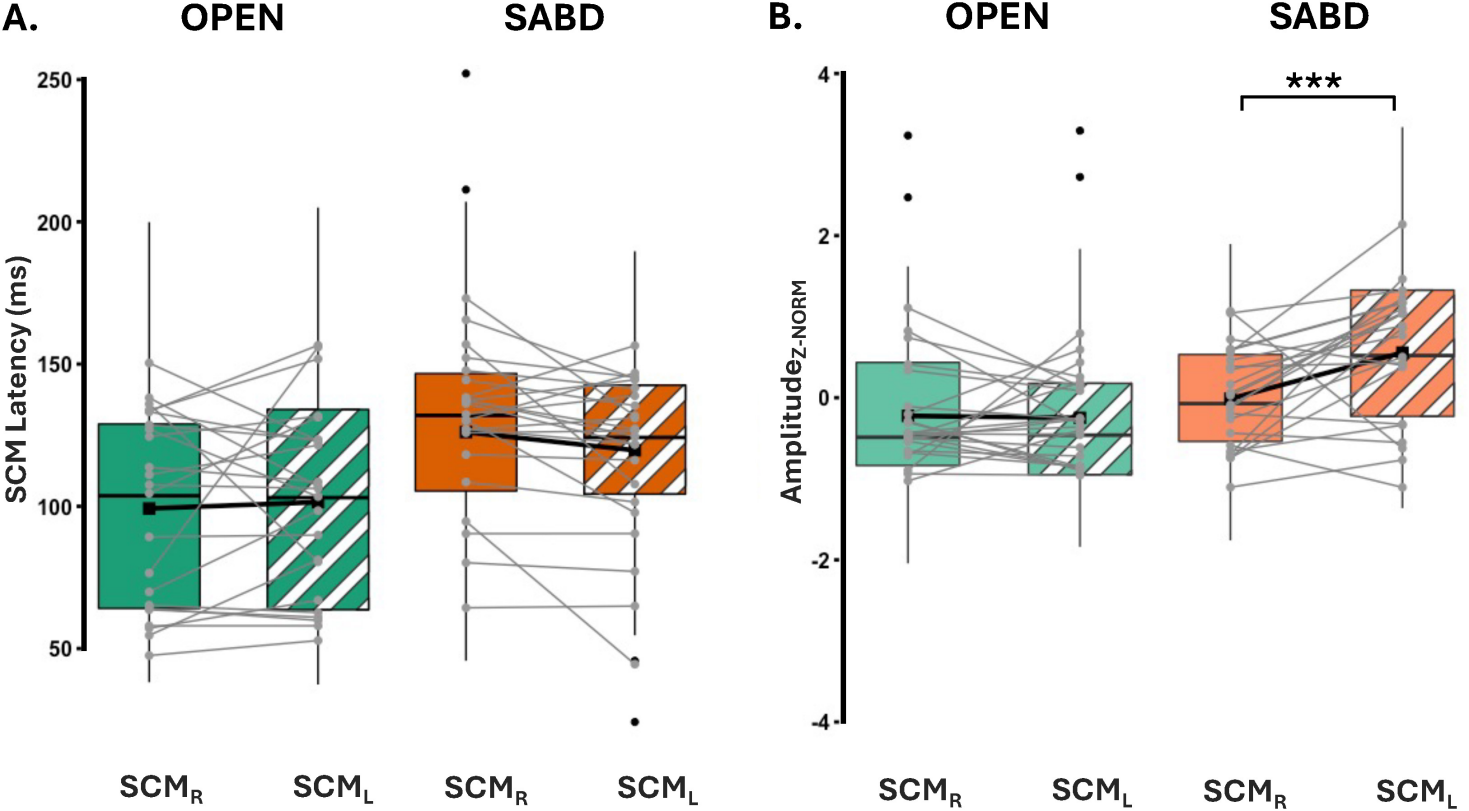
**(A)** Depicts latencies for both right and left SCM. SCM_R_ (solid), SCM_L_ (stripe pattern). **(B)** Illustrates the Z-score normalized SCM amplitude for hand opening (OPEN) (green) and shoulder abduction (SABD) (orange) tasks. Right (solid) and Left (stripe) SCMs. Top, middle, and bottom lines of the box plot indicate the 75th percentile (top quartile), 50th percentile (median), and 25th percentile (bottom quartile), respectively. Black connecting squares in box represent the arithmetic mean. Round gray connecting dots represent the mean of each participant while the black dots are outliers. *n* = 23. ***p < 0.0001.

Figure 5A represents the latencies for both right (SCM_*R*_ - solid) and left (SCM_*L*_ - patterned) SCMs in the SR+ condition. Statistical analysis reported a significant main effect of the Task; SCM activation was significantly delayed in the SABD task compared to the OPEN task, across both Sides : [F(1,230) = 12.9; *p* < 0.001]. However, there was no significant main effect of Side [F(1,368) = 0.5; *p* = 0.46], or an interaction effect between Task and Side [F(1,368) = 3.0; *p* = 0.08]. Contrast analyses of the LME model showed that (1) There were significant delays in both right and left SCM activation in the unilateral SABD compared to the OPEN task (*p* < 0.001 and *p* = 0.02, respectively); (2) Within-task comparison showed that in the OPEN task, there was no significant difference between the right and left SCM latencies (*p* = 0.46); and (3) In the SABD task, the delay observed in the right SCM latency relative to left, did not reach significance (*p* = 0.09, close to significant, See Table 2 middle part).

Figure 5B illustrates the z-score normalized SCM amplitudes for the OPEN and SABD tasks. There was a significant main effect of Task [F(1, 55) = 30.8; *p* < 0.0001], and Side [F(1,400) = 9.4; *p* < 0.001], as well as a significant interaction effect between Task and Side [F(1,400) = 11.2; *p* < 0.001]. *Post hoc* within-task comparison revealed a significant reduction in the right SCM amplitude compared to the left SCM in the SABD task (*p* < 0.0001) but not in the OPEN task (*p* = 0.84; See Table 2 bottom part).

## Discussion

Our main finding was that increasing asymmetrical head/neck postural demands from low (OPEN task) to high (SABD task) significantly delayed bilateral SCM reflexive activation beyond the conventional 120 ms cutoff. Additionally, we observed a reduction in the amplitude of the SCM ipsilateral to the action arm relative to the contralateral side. This posture-dependent modulation of SCM reflexive activation occurred without any difference in the probability of eliciting a true startle (SR+) response when using our SCM latency ≤ 150 ms criteria to classify SR+ trials. This suggests that the startling stimulus likely triggered the reticular formation with similar probability in both tasks. However, given the need for anticipatory postural adjustments (APAs) in preparation for the unilateral SABD task, we provide, for the first time, evidence of posture-dependent modulation of SCM activation in startReact paradigms. Furthermore, we introduce a novel methodology to classify RST-mediated responses irrespective of head/neck postural demands, advancing our understanding of startle responses in varying motor contexts.

### Delayed SCM latency may result from APAs

The observed delay in SCM activation during the SABD task compared to the OPEN task (Figure 2) likely indicates an effect of APAs on SCM reflexive activation (Figure 5A). Since the startle stimulus was delivered before the release of the pre-planned motor task, any adjustments in preparation for the rapid involuntary task release must also occur before the task release. APAs, which preemptively modulate postural muscle activity in anticipation of destabilizing forces, are pre-programmed based on prior knowledge of the movement’s effects on posture and balance (Caronni and Cavallari, 2009a,b; Zattara and Bouisset, 1986). In our study, participants were aware of the task in advance and experienced the same task at least three times in the beginning of each block. Given that unilateral SABD task requires the preservation of neck posture (Falla et al., 2004), APAs may modulate the SCM excitability to facilitate the neck stability.

Accordingly, when APA requirements are low, we do not anticipate a significant impact on SCM reflexive activation. This likely explains why previous startReact studies focusing on elbow, wrist, and finger tasks (Honeycutt et al., 2013; Carlsen et al., 2009) did not report significant task differences in SCM latency, since these tasks do not substantially alter neck muscle activation required for postural preservation. For instance, I.B.M. Van Der Fits et al. (1998) demonstrated that driving the shoulder abductor muscle has a more significant impact on neck/shoulder/trunk postural muscles compared to driving the biceps in fast arm movements. Similarly, bilateral SABD, which imposes fewer postural challenges than unilateral SABD (Yamane et al., 2022) due to the balancing forces and moments between both arms, did not show significant task-related differences in SCM latency (Maslovat et al., 2023). This is further supported by findings that unilateral shoulder movements elicit more pronounced APAs than bilateral ones, as evidenced by greater asymmetries in muscle activation prior to movement onset (Bouisset and Zattara, 1988).

Additionally, our results are consistent with a previous study from Valls-Solé et al. (1999), who observed an increase in the mean SCM latency upon SAS in standing participants performing a fast rise on the tiptoes compared to sitting participants performing wrist flexion or extension movements. In contrast, Brown et al. (1991a) found no significant difference in SCM latency between static sitting and standing alone. The delay in SCM reflex activation, observed only when postural demands increased during a planned movement rather than in a static posture, suggests that while SCM reflexive activation can be triggered by SAS, its modulation depends on the impact of the planned movement on asymmetrical postural demands.

### A data-driven approach for classifying RST-mediated responses irrespective of head/neck postural demands

In startReact paradigms, SCM latencies less than 120 ms following an SAS are widely accepted to indicate RST-mediated startle response, though this threshold lacks strong empirical support (Carlsen et al., 2007). Our results in Figures 2C, D suggest that adhering strictly to this 120 ms cutoff might overlook significant RST-mediated responses, given the increased likelihood of STARTLE trials under the SABD task exceeding the 120 ms cutoff. As a result, trials that are still RST-mediated can be incorrectly labeled as SR−, producing an apparent difference in SR+ probability between tasks. This misclassification is at odds with the well-established bias of the RST toward proximal muscles (Lawrence and Kuypers, 1968; Davidson and Buford, 2006) and highlights the limitations of a fixed threshold. To address this, we adopted a data-driven approach for determining the SCM latency cutoff. Although this method did not reveal significant task differences in SR+ probability, it preserved robust differences in premotor RT, which is a more reliable physiological marker of RST involvement (Maslovat et al., 2021).

Our approach is the first to leverage the physiological differences between true startle-induced SCM reflexes and task-induced SCM activation, both of which contribute to neck stabilization. Their difference exists in that true startle-induced SCM reflexes are released via accessory neurons, independently of the RST-mediated response (Maslovat et al., 2012; Tapia et al., 2022); whereas task-induced SCM activation is likely a postural adjustment mediated by cortical processing in response to neck postural perturbations caused by the task. We thus proposed that task-induced SCM activation aligns more closely with the onset of the primary muscle activation, whereas true-startle-induced SCM reflex operates more independently of primary muscle onset. To capture this relationship, we modeled it using a sigmoid function (Figure 3).

We confirmed the robustness of our novel approach by demonstrating that both OPEN and SABD tasks are susceptible to the startReact, consistent with literature (Honeycutt et al., 2013; Maslovat et al., 2023; Davidson et al., 2007). Importantly, the primary reaction time (RT) results revealed a significantly larger difference between true and false startle responses in the SABD task compared to the OPEN task. This finding aligns with the proximal-to-distal gradient of reticulospinal tract (RST) projections (Lawrence and Kuypers, 1968), further demonstrating the sensitivity of our method in detecting task-dependent variations in startReact effect.

This data-driven approach is particularly advantageous in contexts with complex postural demands or different startReact modalities that extend beyond acoustic stimulation. For instance, Daher et al. (2024) demonstrated that a startling electric stimulus can also elicit a SR+ response accompanied by SCM reflexive activation. However, the SCM latency was significantly delayed beyond the traditional 120 ms compared to that triggered by SAS. Similarly, Ravichandran et al. (2013) showed that a startling postural perturbation triggered prior to the release of a planned elbow extension elicited both SCM reflexive activation and rapid involuntary elbow extension. Yet, the SCM reflexive activation was significantly delayed relative to that triggered by a SAS. These studies highlight the need for data-driven methods to categorize SR+ responses across different sensory modalities. Additionally, a data-driven method can also better accommodate population-specific differences, such as those seen in stroke survivors (Honeycutt et al., 2015; Lee et al., 2022) and Parkinson’s disease patients (Nonnekes et al., 2014), who often exhibit distinct reaction time profiles.

In summary, the < 120 ms threshold may still be appropriate for SAS-triggered movements that have minimal or no APAs, where SCM reflexes remain consistent across trials. For example, when we analyzed the OPEN task alone, the sigmoid fit yielded a cutoff of ∼128 ms, which aligns with the traditional criterion. However, in conditions where the startle reflex may be altered e.g., tasks with increased postural demands or in clinical populations with altered brainstem excitability (Sangari and Perez, 2019; Mooney et al., 2024), we recommend using a data-driven approach, tailored to the characteristics of the dataset. In this study, we implemented a sigmoid fit to model the relationship between SCM latency and reaction time in STARTLE trials, using the inflection point to classify reflexive (SR+) versus task-induced responses. We deliberately included both OPEN and SABD data in this fit to derive a classification threshold that generalizes across tasks. Nevertheless, we recognize that the appropriateness of the sigmoid fit can vary by task. In SABD, where voluntary RT and SCM onset are more tightly linked due to postural demands, the characteristic sigmoid shape may be obscured, and alternative approaches [e.g., relative latency (voluntary RT–SCM latency) or coherence analysis (Grosse and Brown, 2003)], may provide clearer separation. Notably, although the OPEN-only analysis yielded a cutoff closer to the traditional 120 ms threshold, applying a 120 ms cutoff for OPEN and a 150 ms cutoff for SABD did not alter the observed task differences. This suggests that the data-driven method reliably distinguished true versus false startle responses.

### Contralateral bias of inhibitory APAs and reduced right SCM amplitude in unilateral SABD support APA-induced suppression under asymmetrical postural demands

The observed suppression in right SCM amplitude (contralateral with respect to the cortex) compared to left (ipsilateral) SCM muscles in the SABD task cannot be attributed to differences in hearing thresholds between the two ears, as no significant difference was observed (Table 1). Instead, these results are likely explained by the contralateral bias in cortical input to the SCM and contralateral cortical descending drive of inhibitory APAs causing the suppression (Caronni and Cavallari, 2009b; Caronni and Cavallari, 2009a).

Physiological evidence using transcranial stimulations demonstrates that although the SCM receives bilateral cortical inputs, in neurologically intact participants, the contralateral cortical inputs are faster and stronger than the ipsilateral ones. (Thompson et al., 1997; Berardelli et al., 1991; Gandevia and Applegate, 1988). Additionally, deep brain stimulation of the subthalamic nucleus consistently triggers greater peak-to-peak amplitude in the contralateral SCM via corticobulbar axons (Costa et al., 2007), reinforcing the contralateral bias in cortical projections to the SCM.

When APA impact is low, such as during OPEN task, the SAS-induced SCM reflex is generally considered bilateral, as evidenced by the bilateral synchronous SCM response observed following SAS when subjects are relaxed in a chair (Brown et al., 1991b). Thus, the observed symmetry in SCM latency and amplitude during the OPEN task aligns with this bilateral nature of the startle reflex.

### Limitations

We cannot entirely dismiss the possibility that the observed delay in SCM latency may arise as a result of task sequencing. Unlike previous startReact studies that fully counterbalance task order (Drummond et al., 2017), in our study two-thirds of participants began with the OPEN task. Nevertheless, we accounted for the possibility of this confound in our linear mixed model by including the task order as a random effect. Despite the variability introduced by the order, our analysis confirmed a delay in SCM latency during SABD. Therefore, this suggests that the delay is attributable to task-specific demands rather than to differences in task order.

Additionally, we cannot entirely rule out the possibility of reactive postural adjustments in a unilateral SABD task on the SAS-induced SCM activation. APAs are based on the system’s estimation of postural perturbations before the movement, but additional postural adjustments occur during and after movement to correct any deviations (Bouisset and Do, 2008). For example, unilateral right shoulder abduction induces a torsional perturbation of the head and neck toward the left side, leading to significantly greater left SCM activity to compensate for this perturbation. Consistent with this interpretation, Siegmund et al. (2001) observed an increase in the left SCM amplitude in response to a right neck rotation, which may be indicative of a postural adjustment to the rotation perturbation. While Siegmund et al. (2001) calculated SCM amplitude using a larger window based on head acceleration onset, we applied a 100 ms time window following SCM onset to minimize the impact of such reactive postural adjustments while maintaining an acceptable signal-to-noise ratio.

Lastly, we acknowledge that our postural muscle recordings were limited to the bilateral SCMs, reflecting our specific focus on how postural demands influence SCM activation. As SCM primarily functions to support the head and neck posture, our task design intentionally manipulated head/neck postural demands by removing external support of the head and employing a SABD task known to influence head/neck stability (Falla et al., 2004). To isolate these demands, we minimized trunk-related postural adjustments by securing the torso with straps across the shoulders and chest, and providing back support using the Biodex chair. While this approach effectively limited trunk involvement, expanding the set of postural muscles monitored in future studies could enhance our understanding of APAs and their contribution to SCM activity.

## Conclusion

We demonstrated that with increased postural demands from hand opening to shoulder abduction, the SCM activation in startReact is delayed, with a significantly reduced contralateral SCM reflexive activation observed only in the SABD task. This reduction suggests that under conditions where head and neck posture is asymmetrically perturbed, inhibitory anticipatory postural adjustments suppress the SCM reflex. Our findings challenge the conventional 120 ms SCM latency cutoff commonly used to classify startle responses as indicative of reticulospinal drive in all tasks. In response, we propose a data-driven method that models the relationship between SCM latency and reaction time using a sigmoid function, offering a more physiologically grounded threshold for identifying true startle responses, under increased postural demands. This advancement is crucial for the understanding of reticulospinal contributions to motor control.

## Data Availability

The original contributions presented in this study are included in this article/supplementary material, further inquiries can be directed to the corresponding author.

## References

[B1] Baker S. N. (2011). The primate reticulospinal tract, hand function and functional recovery. *J. Physiol.* 589 5603–5612. 10.1113/jphysiol.2011.215160 21878519 PMC3249036

[B2] Bartsch-Jiménez A. Valero-Cuevas F. J. (2025). Vestibular contribution to motor output is also suppressed by voluntary action of the arm. *J. Physiol.* 603 2699–2711. 10.1113/JP287077 40178515

[B3] Berardelli A. Priori A. Inghilleri M. Cruccu G. Mercuri B. Manfredi M. (1991). Corticobulbar and corticospinal projections to neck muscle motoneurons in man. A functional study with magnetic and electric transcranial brain stimulation. *Exp. Brain Res.* 87 402–406. 10.1007/BF00231857 1769390

[B4] Bhardwaj N. Yadala S. (2023). *Neuroanatomy, corticobulbar tract.* Treasure Island, FL: StatPearls Publishing. 32310351

[B5] Blouin J. S. Descarreaux M. Bélanger-Gravel A. Simoneau M. Teasdale N. (2003). Self-initiating a seated perturbation modifies the neck postural responses in humans. *Neurosci. Lett.* 347 1–4. 10.1016/s0304-3940(03)00632-3 12865127

[B6] Bouisset S. Do M. C. (2008). Posture, dynamic stability, and voluntary movement. *Neurophysiol. Clin.* 38 345–362. 10.1016/j.neucli.2008.10.001 19026956

[B7] Bouisset S. Zattara M. (1988). “Anticipatory postural adjustments and dynamic asymmetry of voluntary movement,” in *Stance and motion: Facts and concepts*, eds Gurfinkel V. S. Ioffe M. E. Massion J. Roll J. P. (Boston, MA: Springer US).

[B8] Brown P. Rothwell J. C. Thompson P. D. Britton T. C. Day B. L. Marsden C. D. (1991b). New observations on the normal auditory startle reflex in man. *Brain* 114 1891–1902. 10.1093/brain/114.4.1891 1884184

[B9] Brown P. Day B. L. Rothwell J. C. Thompson P. D. Marsden C. D. (1991a). The effect of posture on the normal and pathological auditory startle reflex. *J. Neurol. Neurosurg. Psychiatry* 54 892–897. 10.1136/jnnp.54.10.892 1744643 PMC1014574

[B10] Carlsen A. N. (2015). A broadband acoustic stimulus is more likely than a pure tone to elicit a startle reflex and prepared movements. *Physiol. Rep.* 3:e12509. 10.14814/phy2.12509 26311832 PMC4562592

[B11] Carlsen A. N. Chua R. Inglis J. T. Sanderson D. J. Franks I. M. (2004). Can prepared responses be stored subcortically? *Exp. Brain Res.* 159 301–309. 10.1007/s00221-004-1924-z 15480608

[B12] Carlsen A. N. Chua R. Inglis J. T. Sanderson D. J. Franks I. M. (2009). Differential effects of startle on reaction time for finger and arm movements. *J. Neurophysiol.* 101 306–314. 10.1152/jn.00878.2007 19005006 PMC2637008

[B13] Carlsen A. N. Dakin C. J. Chua R. Franks I. M. (2007). Startle produces early response latencies that are distinct from stimulus intensity effects. *Exp. Brain Res.* 176 199–205. 10.1007/s00221-006-0610-8 16874516

[B14] Caronni A. Cavallari P. (2009b). Supra-spinal circuits shape inhibitory postural adjustments anticipating voluntary index-finger flexion. *Exp. Brain Res.* 198 19–28. 10.1007/s00221-009-1931-1 19593551

[B15] Caronni A. Cavallari P. (2009a). Anticipatory postural adjustments stabilise the whole upper-limb prior to a gentle index finger tap. *Exp. Brain Res.* 194 59–66. 10.1007/s00221-008-1668-2 19082820

[B16] Costa J. Valls-Solé J. Valldeoriola F. Rumià J. Tolosa E. (2007). Motor responses of muscles supplied by cranial nerves to subthalamic nucleus deep brain stimuli. *Brain* 130 245–255. 10.1093/brain/awl336 17151002

[B17] Daher E. Maslovat D. Carlsen A. N. (2024). An intense electrical stimulus can elicit a StartReact effect but with decreased incidence and later onset of the startle reflex. *Exp. Brain Res.* 242 2405–2417. 10.1007/s00221-024-06899-4 39136724

[B18] Davidson A. G. Buford J. A. (2006). Bilateral actions of the reticulospinal tract on arm and shoulder muscles in the monkey: Stimulus triggered averaging. *Exp. Brain Res.* 173 25–39. 10.1007/s00221-006-0374-1 16506008

[B19] Davidson A. G. Schieber M. H. Buford J. A. (2007). Bilateral spike-triggered average effects in arm and shoulder muscles from the monkey pontomedullary reticular formation. *J. Neurosci.* 27 8053–8058. 10.1523/JNEUROSCI.0040-07.2007 17652596 PMC6672715

[B20] Drummond N. M. Leguerrier A. Carlsen A. N. (2017). Foreknowledge of an impending startling stimulus does not affect the proportion of startle reflexes or latency of StartReact responses. *Exp. Brain Res.* 235 379–388. 10.1007/s00221-016-4795-1 27738717

[B21] Falla D. Rainoldi A. Merletti R. Jull G. (2004). Spatio-temporal evaluation of neck muscle activation during postural perturbations in healthy subjects. *J. Electromyogr. Kinesiol.* 14 463–474. 10.1016/j.jelekin.2004.03.003 15165596

[B22] Gandevia S. C. Applegate C. (1988). Activation of neck muscles from the human motor cortex. *Brain* 111 801–813. 10.1093/brain/111.4.801 3401684

[B23] Grosse P. Brown P. (2003). Acoustic startle evokes bilaterally synchronous oscillatory EMG activity in the healthy human. *J. Neurophysiol.* 90 1654–1661. 10.1152/jn.00125.2003 12750424

[B24] Heckman R. L. Perreault E. J. (2019). Uncertainty in when a perturbation will arrive influences the preparation and release of triggered responses. *Exp. Brain Res.* 237 2353–2365. 10.1007/s00221-019-05592-1 31292693 PMC8407195

[B25] Honeycutt C. F. Kharouta M. Perreault E. J. (2013). Evidence for reticulospinal contributions to coordinated finger movements in humans. *J. Neurophysiol.* 110 1476–1483. 10.1152/jn.00866.2012 23825395 PMC4042417

[B26] Honeycutt C. F. Tresch U. A. Perreault E. J. (2015). Startling acoustic stimuli can evoke fast hand extension movements in stroke survivors. *Clin. Neurophysiol.* 126 160–164. 10.1016/j.clinph.2014.05.025 25002367 PMC4268121

[B27] Kumar S. Narayan Y. Amell T. (2000). Role of awareness in head-neck acceleration in low velocity rear-end impacts. *Accid. Anal. Prev.* 32 233–241. 10.1016/s0001-4575(99)00114-1 10688479

[B28] Kuramochi R. Kimura T. Nakazawa K. Akai M. Torii S. Suzuki S. (2004). Anticipatory modulation of neck muscle reflex responses induced by mechanical perturbations of the human forehead. *Neurosci. Lett.* 366 206–210. 10.1016/j.neulet.2004.05.040 15276248

[B29] Lawrence D. G. Kuypers H. G. (1968). The functional organization of the motor system in the monkey. II. The effects of lesions of the descending brain-stem pathways. *Brain* 91 15–36. 10.1093/brain/91.1.15 4966860

[B30] Lee H. Honeycutt C. Perreault E. (2022). Influence of task complexity on movement planning and release after stroke: Insights from startReact. *Exp. Brain Res.* 240, 1765–1774. 35445354 10.1007/s00221-022-06368-w

[B31] Lingenhöhl K. Friauf E. (1994). Giant neurons in the rat reticular formation: A sensorimotor interface in the elementary acoustic startle circuit? *J. Neurosci.* 14 1176–1194. 10.1523/JNEUROSCI.14-03-01176.1994 8120618 PMC6577542

[B32] MacKinnon C. D. Allen D. P. Shiratori T. Rogers M. W. (2013). Early and unintentional release of planned motor actions during motor cortical preparation. *PLoS One* 8:e63417. 10.1371/journal.pone.0063417 23667613 PMC3646782

[B33] MacKinnon C. D. Bissig D. Chiusano J. Miller E. Rudnick L. Jager C. (2007). Preparation of anticipatory postural adjustments prior to stepping. *J. Neurophysiol.* 97 4368–4379. 10.1152/jn.01136.2006 17460098

[B34] Maslovat D. Franks I. M. Leguerrier A. Carlsen A. N. (2015). Responses to startling acoustic stimuli indicate that movement-related activation is constant prior to action: A replication with an alternate interpretation. *Physiol. Rep.* 3:e12300. 10.14814/phy2.12300 25663524 PMC4393208

[B35] Maslovat D. Kennedy P. M. Forgaard C. J. Chua R. Franks I. M. (2012). The effects of prepulse inhibition timing on the startle reflex and reaction time. *Neurosci. Lett.* 513 243–247. 10.1016/j.neulet.2012.02.052 22387455

[B36] Maslovat D. Sadler C. M. Smith V. Bui A. Carlsen A. N. (2021). Response triggering by an acoustic stimulus increases with stimulus intensity and is best predicted by startle reflex activation. *Sci Rep*. 11:23612. 34880317 10.1038/s41598-021-02825-8 PMC8655082

[B37] Maslovat D. Santangelo C. M. Carlsen A. N. (2023). Startle-triggered responses indicate reticulospinal drive is larger for voluntary shoulder versus finger movements. *Sci. Rep.* 13:6532. 10.1038/s41598-023-33493-5 37085607 PMC10121700

[B38] Mooney R. A. Anaya M. A. Stilling J. M. Celnik P. A. (2024). Heightened reticulospinal excitability after severe corticospinal damage in chronic stroke. *Ann. Neurol.* 97, 163–174. 10.1002/ana.27103 39387284

[B39] Musiek F. E. Shinn J. Chermak G. D. Bamiou D. E. (2017). Perspectives on the pure-tone audiogram. *J. Am. Acad. Audiol.* 28 655–671. 10.3766/jaaa.16061 28722648

[B40] Nonnekes J. Geurts A. C. Nijhuis L. B. Van Geel K. Snijders A. H. Bloem B. R. (2014). Reduced StartReact effect and freezing of gait in Parkinson’s disease: Two of a kind? *J. Neurol.* 261, 943–950. 24609973 10.1007/s00415-014-7304-0

[B41] Ravichandran V. J. Honeycutt C. F. Shemmell J. Perreault E. J. (2013). Instruction-dependent modulation of the long-latency stretch reflex is associated with indicators of startle. *Exp. Brain Res.* 230 59–69. 10.1007/s00221-013-3630-1 23811739 PMC3759548

[B42] Sangari S. Perez M. A. (2019). Imbalanced corticospinal and reticulospinal contributions to spasticity in humans with spinal cord injury. *J. Neurosci.* 39 7872–7881. 10.1523/JNEUROSCI.1106-19.2019 31413076 PMC6774405

[B43] Siegmund G. P. Inglis J. T. Sanderson D. J. (2001). Startle response of human neck muscles sculpted by readiness to perform ballistic head movements. *J. Physiol.* 535 289–300. 10.1111/j.1469-7793.2001.00289.x 11507178 PMC2278755

[B44] Sukal T. M. Ellis M. D. Dewald J. P. (2007). Shoulder abduction-induced reductions in reaching work area following hemiparetic stroke: Neuroscientific implications. *Exp. Brain Res.* 183 215–223. 10.1007/s00221-007-1029-6 17634933 PMC2827935

[B45] Takakusaki K. Takahashi M. Obara K. Chiba R. (2017). Neural substrates involved in the control of posture. *Adv. Robot.* 31 2–23. 10.1080/01691864.2016.1252690

[B46] Tapia J. A. Tohyama T. Poll A. Baker S. N. (2022). The existence of the startreact effect implies reticulospinal, not corticospinal, inputs dominate drive to motoneurons during voluntary movement. *J. Neurosci.* 42 7634–7647. 10.1523/JNEUROSCI.2473-21.2022 36658461 PMC9546468

[B47] Thompson M. L. Thickbroom G. W. Mastaglia F. L. (1997). Corticomotor representation of the sternocleidomastoid muscle. *Brain* 120 245–255. 10.1093/brain/120.2.245 9117372

[B48] Valls-Solé J. Rothwell J. C. Goulart F. Cossu G. Muñoz E. (1999). Patterned ballistic movements triggered by a startle in healthy humans. *J. Physiol.* 516 931–938. 10.1111/j.1469-7793.1999.0931u.x 10200438 PMC2269293

[B49] Van Der Fits I. B. Klip A. W. Van Eykern L. A. Hadders-Algra M. (1998). Postural adjustments accompanying fast pointing movements in standing, sitting and lying adults. *Exp. Brain Res.* 120 202–216. 10.1007/s002210050394 9629962

[B50] Welniarz Q. Dusart I. Roze E. (2017). The corticospinal tract: Evolution, development, and human disorders. *Dev. Neurobiol.* 77 810–829. 10.1002/dneu.22455 27706924

[B51] Wright K. Adjei E. Dewald J. P. A. Yao J. (2024). *Physical therapy and human movement science: Reticulospinal engagement increases as a function of shoulder abduction load. Program No. PSTR120.23. 2024 neuroscience meeting planner.* Chicago, IL: Northwestern University.

[B52] Yamane M. Aoki M. Sasaki Y. Hayashi T. (2022). Feedforward coactivation of trunk muscles during rapid shoulder movements. *JSES Int.* 6 660–668. 10.1016/j.jseint.2022.04.003 35813146 PMC9264006

[B53] Yeomans J. S. Frankland P. W. (1995). The acoustic startle reflex: Neurons and connections. *Brain Res. Brain Res. Rev.* 21 301–314. 10.1016/0165-0173(96)00004-5 8806018

[B54] Zattara M. Bouisset S. (1986). Chronometric analysis of the posturo-kinetic programming of voluntary movement. *J. Mot. Behav.* 18 215–223. 10.1080/00222895.1986.10735378 15136280

